# Modulating the Structure of EGFR with UV Light: New Possibilities in Cancer Therapy

**DOI:** 10.1371/journal.pone.0111617

**Published:** 2014-11-11

**Authors:** Manuel Correia, Viruthachalam Thiagarajan, Isabel Coutinho, Gnana Prakash Gajula, Steffen B. Petersen, Maria Teresa Neves-Petersen

**Affiliations:** 1 Department of Physics and Nanotechnology, Aalborg University, Aalborg, Denmark; 2 BioPhotonics Group, Department of Nanomedicine, International Iberian Nanotechnology Laboratory (INL), Braga, Portugal; 3 School of Chemistry, Bharathidasan University, Tiruchirappalli, India; 4 Department of Health Science and Technology, Aalborg University, Aalborg, Denmark; 5 The Institute for Lasers, Photonics and Biophotonics, University at Buffalo, The State University of New York, New York, United States of America; University-Hospital of Parma, Italy

## Abstract

The epidermal growth factor receptor (EGFR) is a member of the ErbB family of receptor tyrosine kinases. EGFR is activated upon binding to e.g. epidermal growth factor (EGF), leading to cell survival, proliferation and migration. EGFR overactivation is associated with tumor progression. We have previously shown that low dose UVB illumination of cancer cells overexpressing EGFR prior to adding EGF halted the EGFR signaling pathway. We here show that UVB illumination of the extracellular domain of EGFR (sEGFR) induces protein conformational changes, disulphide bridge breakage and formation of tryptophan and tyrosine photoproducts such as dityrosine, N-formylkynurenine and kynurenine. Fluorescence spectroscopy, circular dichroism and thermal studies confirm the occurrence of conformational changes. An immunoassay has confirmed that UVB light induces structural changes in the EGF binding site. A monoclonal antibody which competes with EGF for binding sEGFR was used. We report clear evidence that UVB light induces structural changes in EGFR that impairs the correct binding of an EGFR specific antibody that competes with EGF for binding EGFR, confirming that the 3D structure of the EGFR binding domain suffered conformational changes upon UV illumination. The irradiance used is in the same order of magnitude as the integrated intensity in the solar UVB range. The new photonic technology disables a key receptor and is most likely applicable to the treatment of various types of cancer, alone or in combination with other therapies.

## Introduction

The ErbB family of receptor tyrosine kinases (RTKs) plays a key role in regulating normal cellular processes such as cell survival, proliferation and migration [1], [2] and have a critical role in the development and progression of cancers [3]. The epidermal growth factor receptor (EGFR; ErbB1) is a member of this family [4]. EGFR binding to ligands such as epidermal growth factor (EGF) or transforming growth factor alpha (TGF-α) leads to receptor dimerization and to the activation of the intracellular tyrosine kinase domain [1], [2]. The extracellular domain of EGFR (sEGFR, soluble extracellular region of EGFR) comprises 4 sub-domains: 2 large homologous binding domains (I and III), and 2 homologous furin-like cysteine rich domains (II and IV). Domains I, II and III have been found to be directly involved in ligand binding and dimer formation that precede the mechanism of signal transduction by RTKs [1], [5], [6]. Cancer progression has been correlated with the increase in the number of EGFR molecules on the cell surface [7]. High expression of EGFR is generally associated with invasion, metastasis, late-stage disease, chemotherapy resistance, hormonal therapy resistance and poor general therapeutic outcome. EGFR overexpression has been found to be a strong prognostic indicator in head and neck, ovarian, cervical, bladder and oesophageal cancers, a modest prognostic indicator in breast, colorectal, gastric and endometrial cancer and a weak prognostic indicator in non-small-cell lung cancer [7]. EGFR is the target of many chemotherapeutical approaches because EGFR activation results in cell signaling cascades that promote tumor growth. Inhibition of EGFR function is therefore a rational treatment approach. Typical chemotherapeutical agents are EGFR tyrosine kinase inhibitors which compete with ATP at the intracellular tyrosine kinase domain, and monoclonal antibodies (mAbs) that prevent ligand-binding or receptor dimerization. Blocking the binding of EGF to EGFR can abolish cancer proliferation, invasion, metastasis, angiogenesis and inhibition of apoptosis [8].

We have previously reported that UVB illumination (280 nm, 0.35 W/m^2^ for 30 min) of cancer cells overexpressing EGFR led to the arrest of the EGFR signaling pathway [9]. Proof-of-concept has been documented on cell lines A431 (human epidermoid carcinoma cells) and Cal39 (derived from human vulva squamous cell carcinoma cells). The irradiance used was lower than the total UVB solar irradiance [10]. UVB prevented EGF induced activation of EGFR, abolishing phosphorylation of the EGFR intracellular domain and of other key downstream signaling proteins such as AKT (Protein Kinase B) and the mitogen activated protein kinases (ERK1 and 2). AKT plays a key role in e.g. glucose metabolism, apoptosis, cell proliferation, transcription and cell migration. AKT is involved in cellular survival pathways by inhibiting apoptotic processes [11]–[16]. The ERK kinases act in a signaling cascade that regulates cellular processes such as proliferation, differentiation, and cell cycle progression [17].

**Figure 1:**
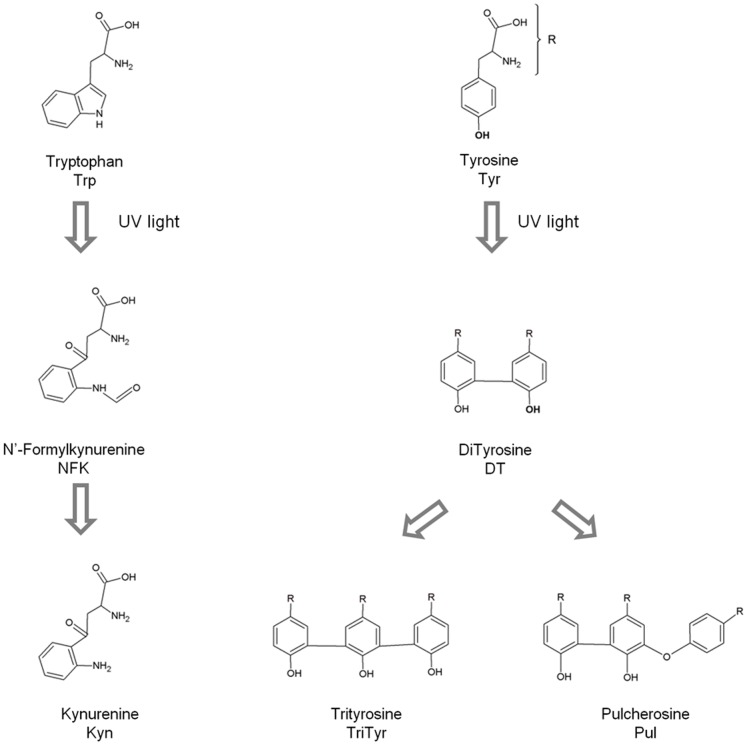
Typical photoproducts generated upon UV illumination of the aromatic residues Trp and Tyr.

**Figure 2:**
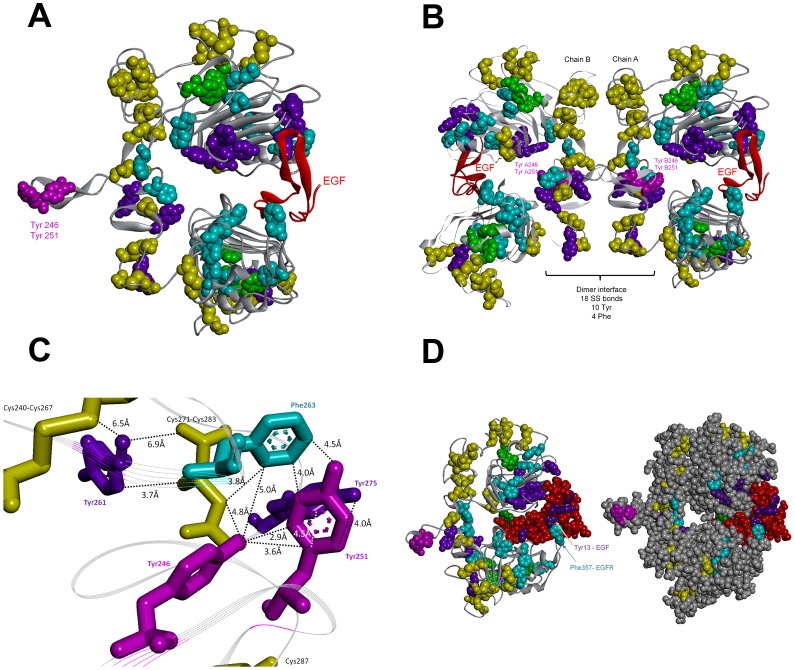
Structural analysis of the EGFR extracellular domain and interactions with EGF upon dimerization. (A) Crystal structure of EGFR extracellular domain (1ivo.pdb, chain A) [1]. The disulphide bridges and aromatic residues atoms are displayed as CPK: SS bridges in yellow, Trp in green, Tyr in violet (Try 246 and Tyr 251 are displayed in pink) and Phe in cyan. Only 18 out of 25 SS bridges, 5 out of 6 Trp residues, 13 out of 16 Tyr residues and 17 out of 18 Phe residues are displayed since some residues are missing in the pdb file. (B) Crystal structure of a 1∶1 complex between human EGF and the dimeric form of EGFR extracellular domain (1ivo.pdb, chain A and B and 2 EGF molecules [1]). EGF is displayed in red. (C) Zoom into interface region present in the dimeric of EGFR (extracellular domains). Distances among some of the aromatic residues and the nearby disulphide bridges are also displayed. (D) Crystal structure of a 1∶1 complex between human EGF and the dimeric form of EGFR extracellular domain. (1ivo.pdb, chain A and B and 2 EGF molecules [1]) EGF is shown in red. In the right panel all atoms are displayed as CPK. EGF docking to sEGFR involves extensive non-covalent contacts.

One of the possible reasons for the observed UV light induced arrest of the EGFR signaling pathway is the UVB induced photochemistry, leading to conformational changes in EGFR which most likely prevent the correct binding to EGF. Our previous work on UVB induced photochemistry in proteins (wavelengths used 275–295 nm) [18]–[22] supports this hypothesis. UVB excitation of aromatic residues in proteins leads to the disruption of disulphide bridges and to the formation of photoproducts, such as N-formylkynurenine (NFK), kynurenine (Kyn) [23], [24] and dityrosine (DT) [25]–[27] (see Figure 1). Such reactions will most likely induce structural changes in proteins which may impair their activity [22]. Proteins that are rich in aromatic residues and disulphide bridges are likely to have their structure considerably impaired upon prolonged UVB excitation. sEGFR is extremely rich in disulphide bridges when compared to the natural average abundance of disulphide bridges in protein structures of the size of sEGFR, as will be shown in the results section. The % of disulphide bridges in sEGFR is approximately 13 times higher than expected for a protein of its size. The expected average results have been previously reported by our group after a comprehensive analyses of the features of the disulphide bridges present in 131 non-homologous single chain protein structures [28]. Furthermore, the extracellular domain of sEGFR has 40 aromatic residues and 25 disulphide bridges per monomer and many of the aromatic residues are in close proximity of disulphide bridges (see Figure 2). Therefore, it is likely that UV illumination of this protein will lead to disulphide bridge breakage and to photoproducts. This hypothesis will be tested in our present paper.

Whereas the dangers of overexposure to sunlight have been well publicized, less attention has been given to the health benefits of UV-exposure. The effects of UV light on biomolecules, cells and tissues will depend on the energy delivered per unit area. UVB (280–315 nm) radiation is the primary contributor to vitamin D production, which has a protective effect in colon, prostate, and breast cancer [29], [30]. Exposure to sunlight in low doses has also been linked to improved cancer survival rates [31], [32]. UV light has been used to successfully treat rickets, psoriasis, lupus vulgaris, vitiligo, atopic dermatitis and localized scleroderma and jaundice (World Health Organization. The known health effects of UV. *Ultrav. Radiat. INTERSUN Program. - FAQ* at http://www.who.int/uv/faq/uvhealtfac/en/index1.html). There is no direct and conclusive evidence to suggest an increased risk of skin cancer from UVB treatments for psoriasis if radiation doses are respected. UV light is currently being used to treat cutaneous T-cell lymphoma (American Cancer Society, 2011 at http://www.cancer.org). This therapy has been improved by the Food and Drug Administration (FDA, USA). The protective effects of regular weekend sun exposure have been reported, particularly in the case of limb tumors [33]. Melanoma is more frequent among people with indoor occupations than among people having outdoor activities (without sunburn) such as farmers, fishermen, and kids that play outdoors [34], [35]. Skin cancer is still on the increase but this is a result of exposure to UV light both by acute overdosing (causing sunburn) and lifelong cumulative exposure [36]. It is mainly the UVB fraction of solar radiation that gives erythema, melanogenesis (melanin production, which acts as a sunscreen protecting against DNA photodamage and erythema), vitamin D synthesis, and non-melanoma skin cancer, while melanoma is likely to be caused by UVA [37]. Jones et al (1987) found that among the UVA wavelengths, 365 nm had the highest mutagenic effect [38]. UVA fluency rates are several times higher for sunbeds than in the case of direct solar radiation [39]. The above examples document that the health effects of UV exposure are dose and wavelength dependent.

The goal of the present work is to investigate if a low dose of UVB light can induce structural changes in the EGF binding site of EGFR that could impair the correct binding of molecules, such a specific antibody that is known to compete with EGF for EGFR binding. If observed, this will give us insight into why UVB illumination (280 nm) of cancer cells overexpressing EGFR led to the arrest of the EGFR signaling pathway [9]. The UVB irradiance used is in the same order of magnitude as the total irradiance of solar UVB. Fluorescence spectroscopy and circular dichroism studies were carried out in order to detect UVB induced conformational changes. Thermal unfolding studies have been done in order to determine the melting point of the protein prior to and after illumination. Light induced breakage of disulphide bridges has been quantified and the formation of Trp/Tyr photoproducts has been detected. A binding immunoassay was carried out in order to infer the effects of UVB illumination on the structure of the EGF binding site. Our study confirms that low dose UVB (280 nm) illumination of sEGFR induced structural changes in the EGFR binding domain that impaired the binding of a specific antibody known to compete with EGF for EGFR binding to that particular domain. We address the beneficial effects of UV light, its present application in the treatment of diseases and the potential of our putative new photonic therapy for the treatment of localized tumors associated with the overexpression of UV sensitive cellular receptors.

## Results

### Three-dimensional structure of monomeric and dimeric sEGFR

In Figure 2A is displayed the 3D structure of monomeric sEGFR (1ivo.pdb, chain A) bound to Epidermal Growth Factor (EGF, in red). sEGFR contains 25 SS bridges, 6 Trp residues, 16 Tyr residues, and 18 Phe residues, displayed as CPK: SS bridges in yellow, Trp in green, Tyr in violet (Tyr246 and Tyr251 in pink) and Phe in cyan. Only 18 out of 25 SS bridges, 5 out of 6 Trp residues, 13 out of 16 Tyr residues and 17 out of 18 Phe residues are displayed since some residues are missing in the pdb file. Several aromatic residues are located in close spatial proximity of SS bonds.

In Figure 2B is displayed the 3D structure of the EGFR dimer (1ivo.pdb, chains A and B) formed upon binding EGF (in red). The dimer interface is rich in disulphide bridges and aromatic residues (Fig. 2B). Residues Tyr246 and Tyr251 (Figure 2B, in pink) from one chain, are intertwined with the same residues from the other chain. A zoom into one of the chains is displayed in Figure 2C and distances between aromatic residues and disulphide bridges are shown. EGF docking to EGFR is displayed in Figure 2D (EGFR monomer). The interaction between EGFR and EGF appears to be strongly dependent on Van der Waals interactions. Some of those interactions are π-π interactions, such as the interaction between Phe357 (in cyan) of sEGFR and Tyr13 (in violet) of EGF (5 Å).

### Protein Bioinformatics

**Figure 3:**
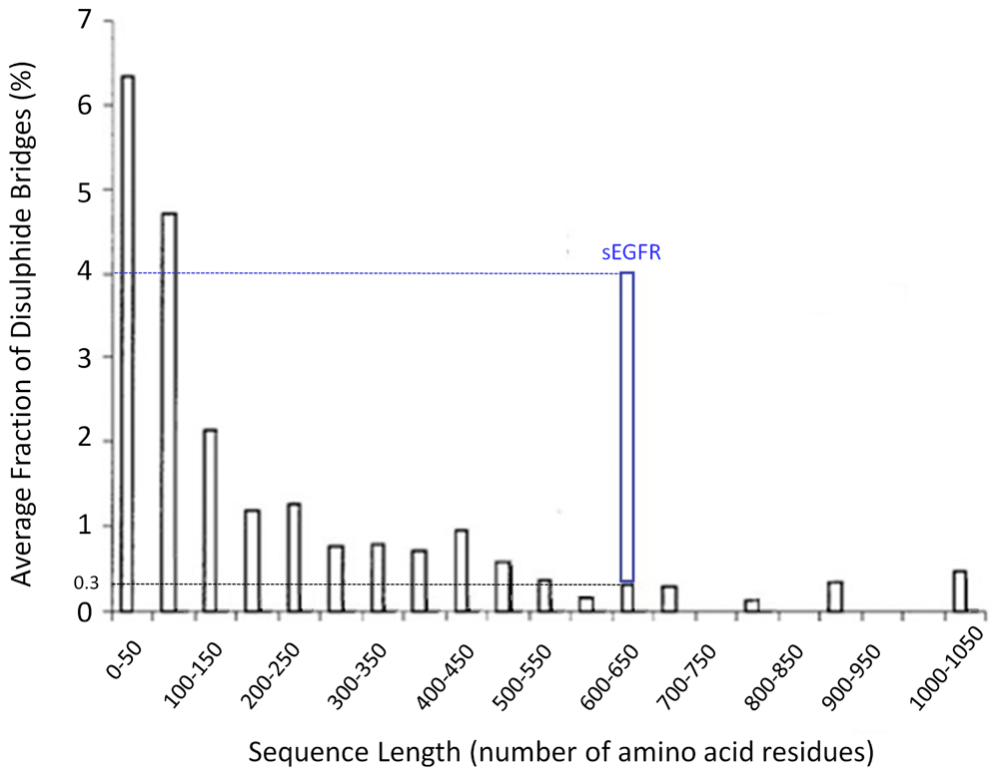
Dependence of the average fraction of disulphide bridges on the protein chain length. The fraction of disulphide bridges was calculated as the number of disulphide bridges found in a protein divided by the protein sequence length (number of amino acids) x 100. The fraction of disulphide bridges present in the monomeric extracellular domain of EGFR is displayed.

In Figure 3 is displayed the fraction of disulphide bridges present in sEGFR and the dependence of the average fraction of disulphide bridges on the protein chain length [28]. The expected average fraction of disulphide bridges for proteins with sequence length of 600–650 residues is ∼0.3%, while in sEGFR (622 aa) it is ∼4%, confirming that this protein is exceedingly rich in disulphide bridges. The expected average results has been previously reported by our group after doing a comprehensive analyses of the features of the disulphide bridges present in 131 non-homologous single chain protein structures [28].

### Steady State Fluorescence

**Figure 4:**
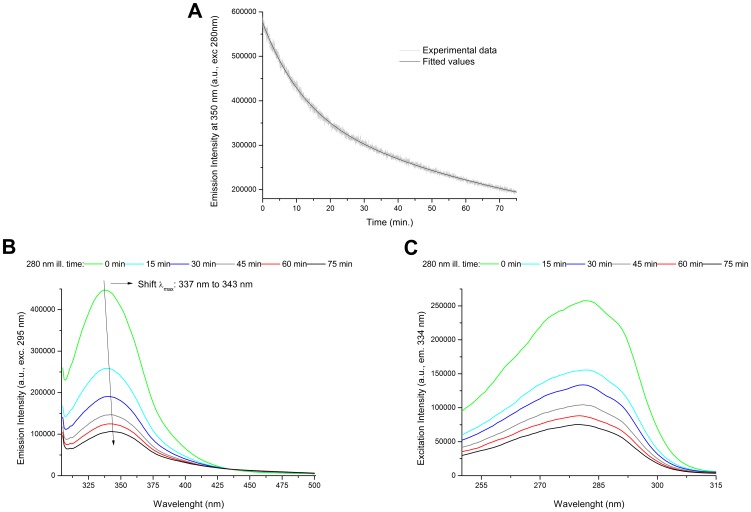
Effects of UV illumination on the intrinsic fluorescence emission of human extracellular EGFR. (A) Fluorescence emission intensity kinetics at 350 nm of human extracellular EGFR (sEGFR) sample during 75 min UV illumination at 280 nm. (B) Fluorescence emission spectra of sEGFR samples obtained upon 295 nm excitation after 280 nm illumination for 0 min, 15 min, 30 min, 45 min, 60 min and 75 min. (C) Fluorescence excitation spectra of sEGFR samples recorded with emission fixed at 334 nm excitation after 280 nm illumination for 0 min, 15 min, 30 min, 45 min, 60 min and 75 min.

The fluorescence emission intensity of sEGFR at 350 nm decreases upon continuous 280 nm excitation (Figure 4A). The kinetic trace is best fitted by a bi-exponential model (Figure 4A and methods). The root mean square error R^2^ was 0.99774. The values recovered from the fitting for C1 and C2 were 367240.44±1182.23 and 208449.95±1054.90, respectively. The rate constants of fluorescence emission intensity decrease k1 and k2 fitted values were 117.63±0.71 min-1 and 12.20±0.10 min-1, respectively.

#### Emission Spectra (excitation 280 nm and 295 nm)

Emission spectra of sEGFR were recorded upon 280 nm excitation before and after continuous 280 nm illumination (15 min, 30 min, 45 min and 75 min at 2.5 W/m^2^) (not shown). A decrease in the intensity of the fluorescence emission at ∼337 nm, where Trp and Tyr emit, is observed due to continuous illumination. The emission intensity at 337 nm decreases by 43% and 74% after 15 min and 75 min of continuous 280 nm illumination, respectively. The wavelength of the most intense peak centered at 337 nm remains constant.

The emission spectra of sEGFR recorded upon 295 nm excitation before and after of continuous 280 nm illumination (15 min, 30 min, 45 min and 75 min) are shown in Figure 4B. A decrease in the intensity of the fluorescence emission at ∼337 nm, where Trp emits, is observed. The emission intensity of sEGFR at 337 nm decreases by 42% and 77% after 15 min and 75 min of continuous 280 nm illumination, respectively. The wavelength of the most intense peak centered at 337 nm is observed to red-shift to 343 nm after 75 min of illumination.

#### Excitation Spectra (emission 334 nm)

In Figure 4C is displayed the excitation spectra of sEGFR with fluorescence emission fixed at 334 nm, before and after continuous 280 nm illumination of the protein for different time periods: 15 min, 30 min, 45 min, 60 min and 75 min. Trp and Tyr absorption contribute to this spectrum. Continuous 280 nm illumination leads to a progressive decrease in the fluorescence excitation intensity. After 15 min and 75 min of illumination, the excitation intensity at 282 nm decreases by 40% and 71%, respectively. The correspondent normalized excitation spectra (not shown) show no shift in the wavelength at maximum excitation intensity (∼282 nm).

#### Fluorescence based protein thermal unfolding studies

**Figure 5:**
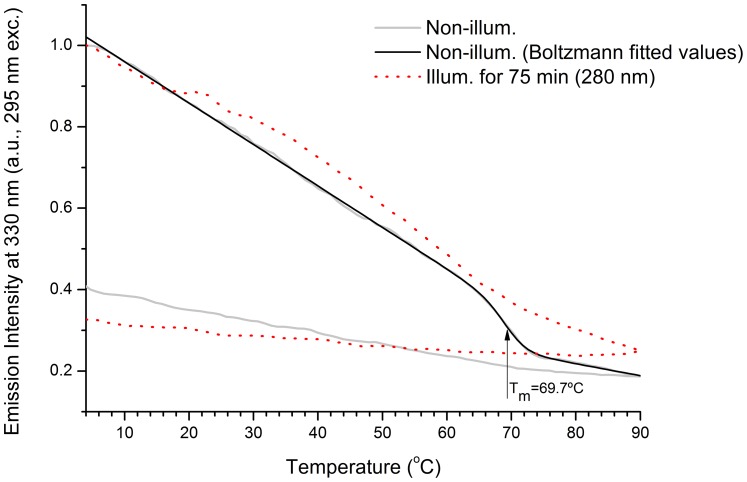
Temperature dependent fluorescence emission intensity at 330 nm upon 295 nm excitation. Non illuminated (non illum.) and UV illuminated (illum. for 75 min) human extracellular EGFR (sEGFR) samples were heated from 4 °C to 90 °C. Fitted values (non-illuminated sample) were obtained using a modified Boltzmann function (see Results section). The transition mid-point recovered from the fitting was at 69.72±0.24°C.

The fluorescence emission intensity at 330 nm (exc. 295 nm) of a fresh sEGFR sample (non-illum.) and illuminated sEGFR sample (75 min at 280 nm) was monitored from 4°C to 90°C and from 90°C to 4°C (see Fig. 5). For both samples the fluorescence emission intensity decreases upon heating. A transition between 65–75°C is observed for the non-illuminated sEGFR. Data has been fitted using a modified Boltzmann function (see methods). The root mean square error for the fitting R^2^ was 0.99774. The values recovered from the fitting for *A*1, *B*1, *A*2, *B*2 and *dx* were 1.06202±0.0014, 0.45482±0.02255, −0.00426±5.41E-04, -0.00296±2.72E-04 and 1.74±0.17, respectively. The recovered parameter *x*0 (69.7°±0.24°C C) corresponds to the mid-point of transition, i.e. to the melting temperature of the protein. This value is in agreement with the inflection point values obtained from the second derivative of the fitted data (not shown). The second derivative is zero (inflection point) within the interval 68.3–69.2°C. Such transition is not observed for the illuminated sample. Furthermore, no transition is observed for both samples when cooling the protein from 90°C to 4°C.

#### Time Resolved Fluorescence

**Figure 6:**
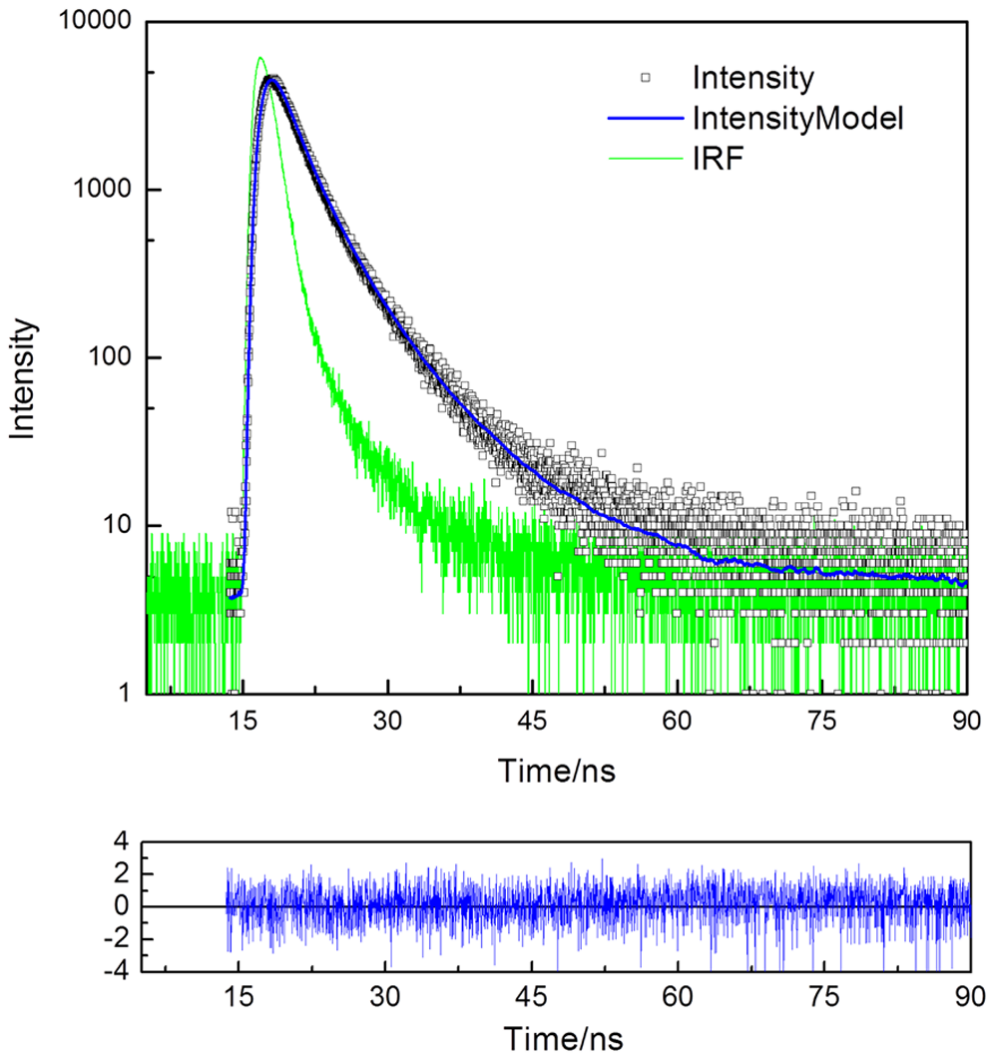
Time-resolved fluorescence measurements. Time-resolved intensity decay data, fitting curve and residuals obtained using the ISS routine for the control sEGFR sample (kept in the dark for 75 min, negative control, NC).

The decay times (τ_i_) and pre-exponential factors (*f*_i_) recovered from the time resolved intensity decays for the control sEGFR sample (75 min in the dark, negative control, NC) and the illuminated sEGFR sample (illum. for 75 min at 280 nm) at pH 7.5 are given in Table 1. Figure 6 shows the experimental data, the fit and residuals assuming three lifetime decays for sEGFR control sample (NC). The value of χ^2^ dropped significantly when progressing from a one lifetime component fit to two and to three components. The fluorescence mean lifetime of sEGFR is shown in Table 1. Non-illuminated sEGFR displays three lifetimes: 1.04 ns, 2.74 ns and 6.23 ns with intensity fractions of 25.6%, 53.1% and 21.1%, respectively. Upon illumination, the contribution of the shortest lived 1.04 ns species increased from 25.6% to 30.6%, the contribution of the 2.74 ns species was kept constant and the contribution of the longer lived species decreased from 21.1% to 16.4%. Furthermore, the lifetime of the longer lived component increased from 6.2 ns to 7.0 ns while the lifetime of the other two components remained constant.

**Table 1:** Lifetimes (τ_i_) and pre-exponential values (α_i_) for sEGFR (negative control and UV illuminated samples) at pH 7.5 obtained by a nonlinear fit (see Figure 6 for example, negative control sEGFR) using the ISS software.

Lifetime (ns)		Intensity fraction		Pre-exponential	
**sEGFR Dark for 75 min (negative control, NC)**					
τ_1_	1.04±0.06	*f*_1_	0.258±0.02	α_1_	24.8
τ_2_	2.74±0.1	*f*_2_	0.531±0.01	α_2_	19.4
τ_3_	6.23±0.2	*f*_3_	0.211 Fixed	α_3_	3.38
<τ> *_f_*_ weighed_	3.04				
**sEGFR Illum. for 75 min (at 280 nm)**					
τ_1_	1.02±0.04	*f*_1_	0.306±0.02	α_1_	30
τ_2_	2.88±0.1	*f*_2_	0.53±0.01	α_2_	18.4
τ_3_	7.0±0.2	*f*_3_	0.164 Fixed	α_3_	2.34
<τ> *_f_*_ weighed_	2.99				

#### Excitation Spectra (emission 400 nm)

**Figure 7:**
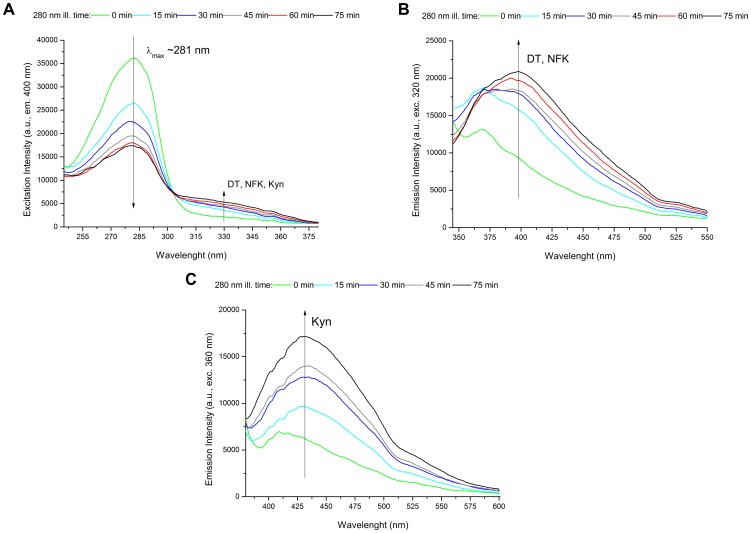
Formation of new fluorescence species and changes in thermal unfolding of the human extracellular EGFR upon UV illumination. (A) Fluorescence emission spectra of human extracellular EGFR (sEGFR) samples recorded upon 320 nm excitation after 280 nm illumination for 0 min, 15 min, 30 min, 45 min, 60 min and 75 min. (B) Fluorescence emission spectra of sEGFR recorded upon 360 nm excitation after 280 nm illumination for 0 min, 15 min, 30 min, 45 min and 75 min. (C) Fluorescence excitation spectra of sEGFR obtained with fluorescence emission fixed at 400 nm after 280 nm illumination for 0 min, 15 min, 30 min, 45 min, 60 min and 75 min.

In Figure 7A are displayed the excitation spectra (emission at 400 nm) obtained before and after continuous 280 nm illumination (0 min, 15 min, 30 min, 45 min, 62 min and 75 min). Spectra were recorded in order to verify the presence of NFK, dityrosine and Kyn (Fig. 1). In Table 2 are displayed their absorbance and fluorescence emission properties. At 0 min, one excitation peak is observed centered at ∼281 nm. Its intensity decays upon UV illumination of sEGFR, decreasing 52% after 75 min. However, illumination of sEGFR leads to an increase of fluorescence excitation above 305 nm. After 75 min, fluorescent excitation intensity increased 115% at 315 nm, where dityrosine maximally absorbs (Abs^max^ at 316 nm, [40], see Table 2), 149% at 322 nm, where NFK maximally absorbs (Abs^max^ at 321 nm, [23], see Table 2) and 173% at 360 nm, where Kyn maximally absorbs (Abs^max^ at 360 nm, [23], see Table 2).

**Table 2:** Absorption and fluorescence spectral characteristics of N-formylkynurenine (NFK), dityrosine (DT) and kynurenine (Kyn) [23], [25], [40].

	Absorption (λ_max_, nm)	Fluor. Em. (λ_max_, nm)
**NKF**	261, 322	400–440^a^
**DT**	284, 316	400–409
**Kyn**	258, 360	434–480^a^

a Depending on solvent characteristics (e.g. polarity).

#### Emission Spectra (excitation 320 nm)

In order to verify the formation of dityrosine (DT) and NFK, emission spectra were obtained upon 320 nm excitation before and after 280 nm illumination (Fig. 7B). An increase in fluorescence emission intensity at 400–405 nm (exc. 320 nm) is observed. The fluorescence emission maximum is centered ∼400 nm, corresponding to the emission maximum of DT (Em^max^ at 400–409 nm, [25], [40], see Table 2) and to a spectral region where NFK can also emit (for NFK, Em^max^ at 400–440 nm [23], Table 2). After 75 min, fluorescence emission intensity at 400 nm increases by 129%. A small emission peak at ∼368 nm is observed in the spectra obtained at 0 min and 15 min of illumination due to the Raman contribution of water. Raman spectral corrections did not completely remove this peak.

#### Emission Spectra (excitation 360 nm)

In order to verify the presence of kynurenine (Kyn) (Abs^max^ at 360 nm, [23], Table 2) in sEGFR, emission spectra were obtained upon 360 nm excitation before and after 15 min, 30 min, 45 min, 60 min and 75 min of 280 nm illumination (Fig. 7C). Kyn absorbs maximally at 360–365 nm and fluoresces maximally at ∼434–480 nm ([23], Table 2). Continuous UVB illumination of sEGFR leads to an increase of fluorescence centered at ∼430–435 nm. The fluorescence intensity at 430 nm increased 53% and 172 after 15 min and 75 min illumination, respectively.

### Thiol group's quantification

**Figure 8:**
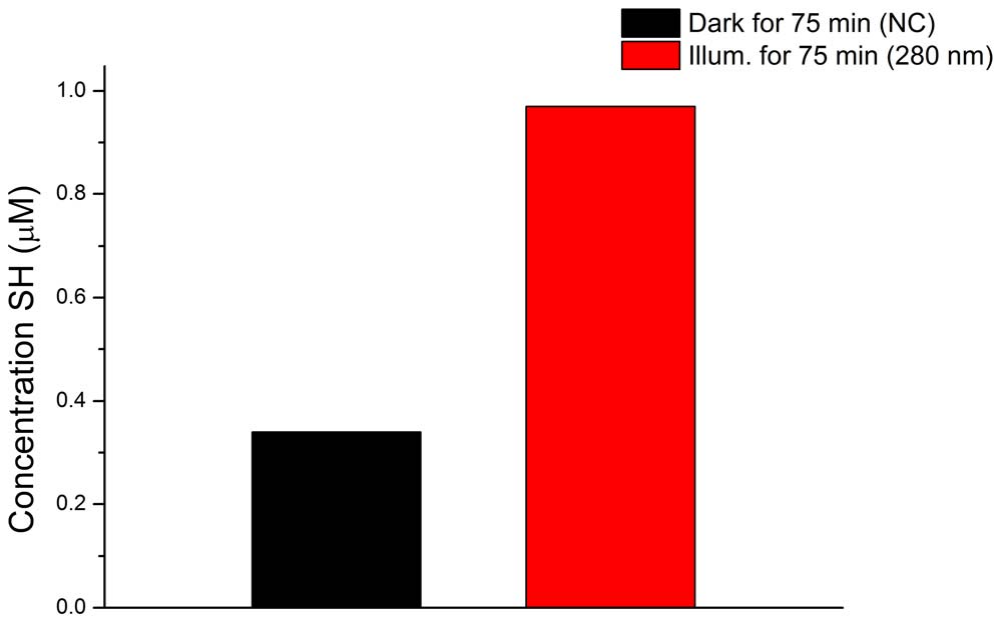
Increase in the concentration of free thiol groups detected in sEGFR upon UV illumination for 75 min. The control sample of sEGFR was kept in the dark for 75 min (negative control, NC). Free thiol groups have been detected using the Ellmann's assay.

In order to confirm that UV illumination of sEGFR has led to SS disruption, the concentration of solvent accessible thiol groups has been determined with the Ellman's assay for a control sEGFR sample kept in the dark for 75 min (negative control, NC) and for a sample previously illuminated at 280 nm for 75 min. The detected concentration of free thiol groups is 2.9 fold higher in the illuminated sample (Fig. 8). Free thiol groups in illuminated sEGFR is ∼1 µM.

### Circular dichroism studies

**Figure 9:**
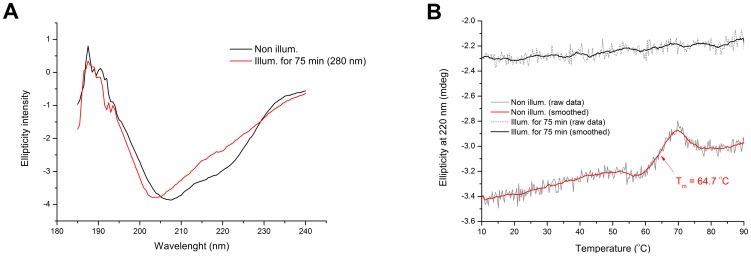
Effect of UVB illumination on the far UV circular dichroism features of sEGFR. (A) Far UV CD spectra were recorded for a fresh sEGFR solution (non illum.) and a sEGFR sample that was previously illuminated at 280 nm (illum. for 75 min). (B) Circular dichroim thermal unfolding curves of fresh sEGFR (non-illum.) and UVB illuminated sEGFR (illum. for 75 min) were obtained upon heating from 4 °C to 90 °C (1 °C.min^−1^). The ellipticity signal was constantly monitored at 220 nm. The melting temperature of non illuminated sEGFR, which corresponds to the transition mid-point of the curve, was recovered upon fitting the curve with a Boltzmann function (see methods).

In Figure 9A are displayed the CD spectra of fresh sEGFR (non-illum.) and illuminated sEGFR (75 min at 280 nm). The far-UV CD spectrum of the non-illuminated sEGFR displays some of the classical far-UV features of protein secondary structure, with the presence of a double minimum at 208–210 nm and 220–222 nm, characteristic of α-helical content. β-sheet structural content (characteristic minimum at ∼218 nm) may also contribute for the second minimum at 220–222 nm [41]. Prolonged excitation with UVB light leads to a decrease in the ellipticity intensity at 205–225 nm and to a spectral shift. The ellipticity at 207.5 nm and at 220 nm has decreased by 9% and 20%, respectively. Furthermore, the first negative peak has shifted from 207.5 nm to 203 nm.

#### Circular dichroism based protein thermal unfolding studies

The ellipticity intensity at 220 nm of fresh sEGFR (non illum.) and of illuminated sEGFR (75 min at 280 nm) was continuously monitored from 4°C to 90°C (Fig. 9B). For both samples the ellipticity intensity at 220 nm decreases upon heating. A transition with mid-point between 60–70 °C is observed for the non-illuminated sEGFR sample. Data has been fitted by a Boltzmann function (see methods). The root mean square error for the fitting R^2^ was 0.99921. The values recovered from the fitting for *A*1, *A*2 and *dx* were −0.96±9.97E-4, −0.83±0.002, and 2.59±0.08, respectively. The temperature of mid-transition *x*0 fitted value was 64.70±0.07 °C, corresponding to the melting temperature of the protein. This value is in agreement with the value recovered by fluorescence spectroscopy displayed in Figure 5. Such transition is not observed for the illuminated sample.

### EGFR binding immunoassay

**Figure 10:**
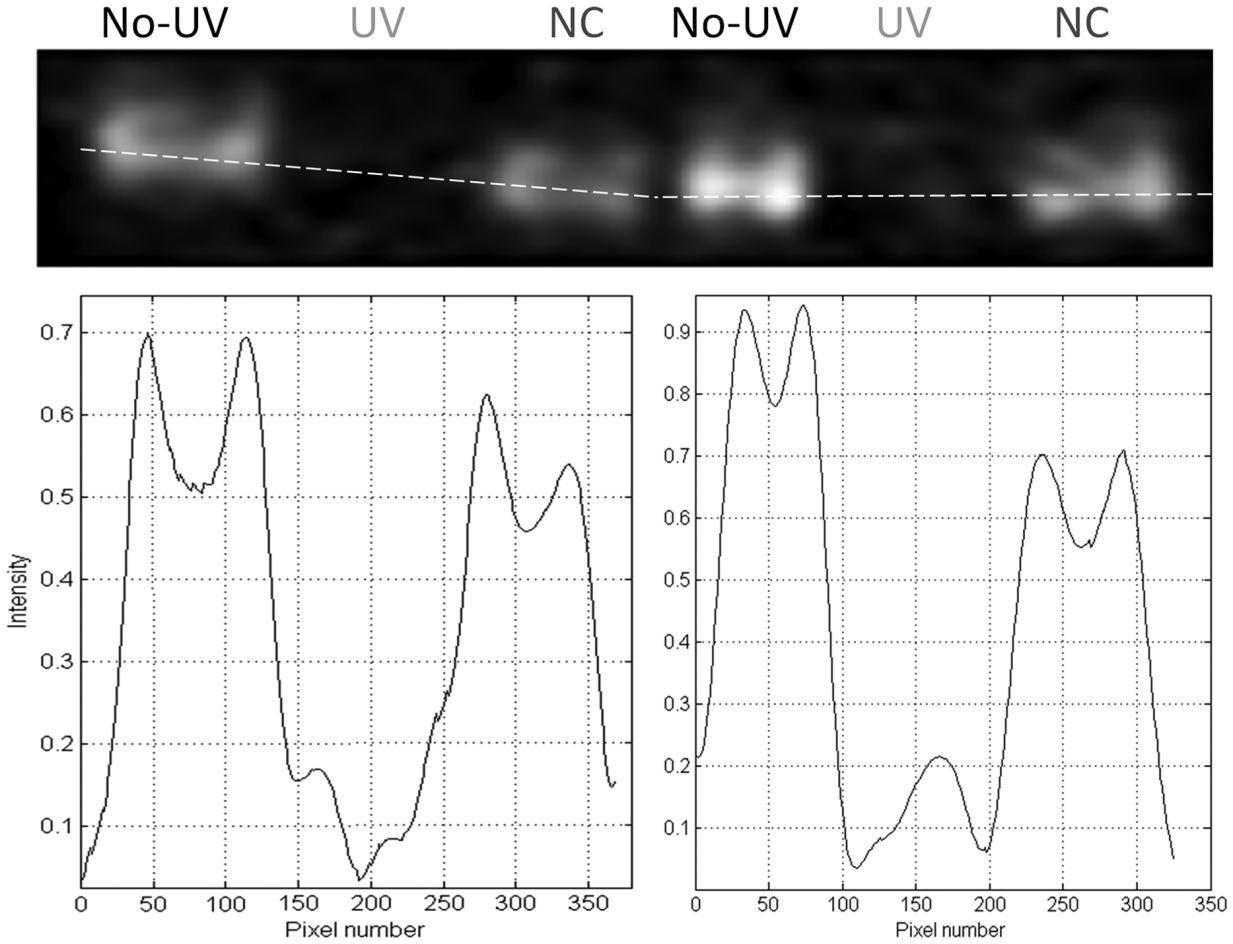
UV illumination of sEGFR prevents binding of anti-EGFR neutralizer antibody LA1 to its target antigen. In each well, exactly 1.4 µg of non illuminated (“No-UV”), UV illuminated for 75 min (“UV”) and negative control (“NC”) sEGFR samples was loaded. Samples loaded on well 1–3 are duplicates of samples 4–6, but were treated independently after UV illumination. The intensity profile along the wells shows that signal is observed in the wells with non-illuminated protein but no signal is present in the wells containing UV illuminated protein samples.

A binding immunoassay was used to indirectly access the effects of UV illumination on the structure of the sEGFR binding site to EGF/TGF-α. Results displayed in Figure 10 show that non-illuminated sEGFR binds LA1 anti-EGFR (lanes “No-UV”, fresh sample; and “NC”, negative control, sample kept in the dark for 75 min). sEGFR sample illuminated with 280 nm light for 75 min no longer binds LA1 anti-EGFR, confirmed by the complete disappearance of the sEGFR band (Figure 10, lanes “UV”). Two sets of duplicate samples were analysed. Signal intensity profiles along the protein bands are shown. The intensity observed in the regions where illuminated sEGFR was present (“UV” lanes) is within the observed noise level (noise intensity from ∼0.02 to 0.2).

## Discussion

UVB excitation of aromatic residues leads to the formation of photoproducts. Tryptophan may form tryptophanyl cation radical, N-formylkynurenine (NKF) and kynurenine (Kyn) (Fig. 1). NFK and Kyn are of particular importance as they are photosensitizers that can generate reactive oxygen species (ROS) upon UV absorption [42], further contributing to protein structural damage. Tyrosine residues are known to be converted into e.g. tyrosil radicals, dityrosine, trityrosine and pulcherosine (Fig. 1). These reactions are likely to lead to changes or to complete loss of protein structure and function [22], [43]. UVB excitation also leads to electron ejection from the side chains of aromatic residues [20], [24], [44]–[46]. The electron can be captured by disulphide bridges, leading to the formation of a transient disulphide electron adduct RSSR^•-^ (see schemes 1–8, [49]), which upon dissociation will form free thiol groups (scheme 9, [47]). Nature has kept aromatic residues in spatial close proximity to disulphide bridges (SS) in proteins [19], [28], making the disruption of SS a likely event upon UV excitation. The schemes below summarize some of the photoproducts formed which contribute to SS disruption, leading to conformation changes that can lead to loss of protein function. For further details please see referenced literature [21], [22], [24].
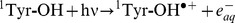
(1)

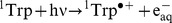
(2)

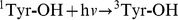
(3)

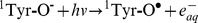
(4)

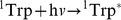
(5)

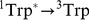
(6)

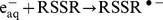
(7)

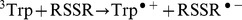
(8)

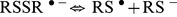
(9)


Proteins rich in aromatic residues and disulphide bridges are most vulnerable to photochemistry and damage. sEGFR is such a protein. It has a total of 6 Trp, 16 Tyr, 18 Phe residues and 25 disulphide bridges (Figure 2A). Interestingly, aromatic residues and disulphide bridges play a critical structural role at the dimer interface (Figures 2B, 2C, and 2D) indicating that UVB induced photochemistry will most likely impair EGFR dimerization. The close proximity between aromatic residues and disulphide bridges (Figure 2C) promotes electron transfer from the aromatic residues to the bridges, leading to their disruption. UV-induced protein inactivation involves fast and short-range electron transfer between photoexcited aromatic residues and nearby disulphide bridges [20], [44], [45], [48]. Zhi Li et al. [49] showed that fast electron transfer is consistent with direct electron transfer between triplet tryptophan and a nearby disulphide bridge, leading to RSSR^•-^ and likely bridge breakage (schemes 8 and 9). As displayed in Figure 3, proteins as large as sEGFR (600–650 aa) are observed to have an average fraction of disulphide bridges no larger than ∼0.3%. This is presumably due to the stabilizing effect of the large hydrophobic core. A short protein has a much smaller hydrophobic core and depends to a larger extent on the structural stability provided by disulphide bridges. An example of this is insulin, a molecule formed by two polypeptide chains containing 21 and 30 residues, respectively, and that has 3 disulphide bridges (SS average fraction of 5.9%). Two disulphide bridges are the only covalent links between the chains. sEGFR displays an average SS fraction of ∼4% compared to the expected ∼0.3%, which means that disulphide bridges in sEGFR are ∼13 times more abundant than expected for a protein of this length. Due to the fact that disulphide bridges are good acceptors of the electrons produced upon UV excitation of the protein, several disulphide bridges might be disrupted during illumination, compromising the structure of sEGFR. Data confirms that UVB illumination of sEGFR leads to the disruption of disulphide bridges (Fig. 8).

Receptors that are not as rich in aromatic residues and disulphide bridges as EGFR is are known be less sensitive of UV light. Huang et al. have reported that PDGF receptors did not appear to be involved in the cells response to UVC illumination, contrary to what they observed with EGFR [50]. They observed that PDGF-B was able to stimulate the tyrosine phosphorylation of the UV illuminated receptor, confirming that the 3D structure of the PDGFR binding domain remained intact after UV illumination. PDGFR lower sensitivity to UV light compared to EGFR might be due to a considerably lower number of disulphide bridges (3 per monomer) and Trp residues (2 per monomer), and lower number of aromatic residues in close spatial proximity to disulphide bridges.

Our previous work on the UV illumination effects on native and W69A mutant cutinase (Trp-depleted mutant) with 280 nm and 295-nm light also documents the crucial interplay between the aromatic residue and the disulphide bridge [51]. Cutinase has a single Trp residue in very close spatial proximity to a disulphide bridge. 280 nm and 295 nm excitation of theTrp residue is known the lead to the disruption of the disulphide bridge mediated by an electron transfer process [20]. This mechanism is severely impaired in the mutant cutinase. In our present study, the ultimate control control would be a sEGFR mutant that did not have aromatic residues and/or disulphide bridges in its structure or at least that did not have aromatic residues in close spatial proximity of disulphide bridges. Those mutants are not available for the simple reason that so many residues would have to be mutated and most certainly the structure of such mutant would no longer be the same as the native structure of the extracellular domain of EGFR. Therefore, studies done with EGFR depleted mutant cells are important in asserting the important of EGFR in UV mediated responses. Evidence is found in literature that strengthens the knowledge that most of the signal pathways involved in UV-induced processes are thought to originate at plasma membrane receptors such as EGFR. Zhang et al. showed that UVA stimulation of the epidermal growth factor receptor (EGFR) may lead to activation of kinases (p70^S6K^/p90^RSK^) through phosphatidyl isositol (PI)-3 kinase and extracellular receptor-activated kinases (ERKs) [52]. Evidence is provided that phosphorylation and activation of p70^S6K^/p90^RSK^ induced by UVA were prevented in Egfr-/- cells (in which basal EGFR expression and its tyrosine phosphorylation induced by UVA were absent) and were also markedly inhibited by EGFR-specific tyrosine kinase inhibitors. Hence, data suggest that UVA-induced p70^S6K^/p90^RSK^ signalling activation is initiated by EGFR-dependent pathways. Furthermore, Xu et al. reported that B82 mouse L cells devoid of EGFR confirmed the key role of EGFR in UVB-dependent signal transduction [53].

Data displayed in Figures 7A, 7B and 7C confirms that UVB illumination of sEGFR leads to the progressive conversion of Trp and Tyr residues into species such as NKF, Kyn and DT. The longer the illumination time the larger such conversion is. Kyn formation is very clear since it absorbs at 360 nm [23] (Table 2). Figure 4B confirms that UVB induces conformational changes in sEGFR due to the observed spectral shift observed upon illumination (solvent relaxation effect). Such red shift is due to the fact that the Trp pool in this protein became more solvent accessible upon illumination. Temperature dependent fluorescence studies (Fig. 5) confirm that illumination of sEGFR induces conformation changes. Prior to illumination a clear thermal transition around 69.7°C was observed. Such transition is due to conformation changes that happen in the Trp moiety of the protein and is associated with changes in solvent accessibility of these residues. The transition is absent if the protein previously has been illuminated for 75 min. This is correlated with the observation that UV illumination renders Trp residues in sEGFR more solvent accessible (Fig. 4B). The cooling experiments have shown that the sEGFR could not refold into its native 3D structure in our *in vitro* experiments. It is also likely that protein aggregation could have occurred upon heating the protein, contributing also to preventing protein refolding. However, the typical formation of a turbid solution associated with protein aggregation was not observed. CD data also confirms that UVB induced protein conformational changes leading to the loss of secondary structural content. As can be seen in Figure 9A, UVB illumination lead to the loss of ellipticity intensity at 207.5 nm (9%) and 220 nm (20%), characteristic of α-helical content and possibly β-sheet structural content. It is likely that the large amount of disulphide bridges in the protein has prevented further loss of ellipticity. The temperature dependent CD data (Figure 9B) shows that non-illuminated sEGFR has a melting temperature around 64.7°C. This value is in good agreement with the value recovered from the temperature dependent fluorescence studies (69.7°C). After 75 min illumination at 280 nm, the observed transition disappears, confirming that UVB has induced protein denaturation. The observed structural changes induced in sEGFR as well as the observed photochemistry are likely causes for the observed changes in fluorescence lifetime distribution in sEGFR (Table 1). Conformational changes that alter the environment surrounding the aromatic residues will most likely induce changes in their lifetimes' distribution. The protein immunoassays displayed in Figure 10 confirm that the UVB illumination of sEGFR induces structural changes in the EGFR binding site to EGF/TGF-α. After illumination, sEGFR no longer binds LA1 anti-EGFR antibody, confirmed by the complete disappearance of the band corresponding to sEGFR (Figure 10). The monoclonal antibody used competes with EGF and TGF-α for binding to EGFR [54]. This demonstrates that UV-illumination of sEGFR compromises the 3D structure of the EGF binding site in the protein. EGF docking to sEGFR is dependent on extensive non-covalent and Van der Vaals interactions between the two molecules, including π-π interactions (see Results, Fig. 2D). It is likely that UV induces conformational changes, disrupting the native tertiary fold that promotes sEGFR-EGF contacts.

The mechanisms through which EGF binds and induces receptor dimerization are not fully understood and the standing model is based on structural studies [1], [55], [56]. Inactive EGFR is kept in its closed conformation via interactions between two sEGFR disulphide rich domains (II and IV) [56]. EGF binding occurs primarily through interactions with domains I and III [1], [55]. Ligand binding requires a change in the relative positions of domains I and III and dimerization occurs upon interaction of subdomains II of two EGFR monomers [1], [5], [55], [56]. It is believed that domain IV has a role in high affinity binding and signal transduction [6]. Considering that the mechanisms of EGF binding and posterior EGFR dimerization can involve all four domains of sEGFR, it is not surprising that UV induced conformational changes will most likely impair binding to EGF. UV induced SS disruption in domain II will likely impair correct EGFR dimerization, but may also affect EGF binding as it is involved in the change of the relative position of domains I and III. Furthermore, domains I and III also contain disulphide bonds, though in lower extent, and SS breakage in these regions can also impair EGF binding. It has also been reported by several groups that EGFR exists as a preformed dimer on the cell-surface [57]. Also in this case, UV induced photochemistry and consequent structural changes in the EGF binding site will most likely impair EGF binding.

Our data confirms that low dose UVB leads to conformational changes in sEGFR, impairing its ability to bind an EGFR specific antibody that competes with EGF for binding EGFR, confirming that the 3D structure of the EGFR binding domain suffered conformational changes upon UV illumination. The present molecular level *in vitro* studies allow us to predict that UV light will most likely also change the structure/function of the extracellular domain of EGFR when present in the cell surface of cancer cells overexpressing EGFR, halting this way EGF-EGFR activation and EGFR dependent key metabolic pathways. Our most recent studies on the UVB (280 nm) illumination of lung cancer cells overexpressing EGFR confirm our predictions (paper in preparation). The present data also supports our previously publish results showing that low dose UVB illumination of cancer cells overexpressing EGFR (A431 and Cal39) led to the arrest of the EGFR signaling pathway [9]. The irradiance used in the present study (2.5 W.m^−2^) and in the previous study (0.35 W.m^−2^) is in the same order of magnitude or lower, respectively, than the total irradiance of sunlight in the UVB region, reported to be 1.75 W.m^−2^ in summer and 0.4 W.m^−2^ in winter (below 313 nm) [10]. The total amount of energy given to the protein solution after 75 min illumination at 280 nm is 90 mJ. This energy is lower than the limit values recommended by the British Photodermatology Group (1000 J, Psoriasis and Psoriatic Arthritis Alliance (PAPAA), 2008, available online at http://www.nhs.uk/ipgmedia/national/psoriasis) in order to prevent cancer. We envision that low dose UVB light can be used as a new photonic therapeutical approach used in order to stop the development of localized cancer, which cells overexpress EGFR or another receptor which structure will be labile to UV light. The treatment could be easily applicable to epidermal skin cancer because UVB light penetrates the skin down to 150–200 µm [37] (online information from the Department of Dermatology School of Medicine, University of California, San Francisco. UV Radiation. at http://www.dermatology.ucsf.edu/skincancer/General/prevention/UV_Radiation.aspx). The thickness of the stratum corneum of our skin is 10–20 µm, the thickness of the epidermis can vary from 50 to 150 µm [37] (online information from the Department of Dermatology School of Medicine, University of California, San Francisco. UV Radiation. at http://www.dermatology.ucsf.edu/skincancer/General/prevention/UV_Radiation.aspx) while the thickness of the dermis varies from 30 to 300 µm (Brannon, H. Skin Anatomy. at http://dermatology.about.com/cs/skinanatomy/a/anatomy.htm). If the localized tumor is located deeper location, UVB light could be delivered to those locations my means of an optical fiber or generated via multiphoton excitation using e.g. IR light. This possible new photonic therapy can also be applied during a surgical intervention.

The role of UV light on the activation or deactivation of EGFR and in promoting cell death or cell survivel remains controversial. We have previously described the attenuation of EGFR signaling as detected by the phosphorylation status of key downstream molecules i.e. AKT and the mitogen activated protein kinases ERK1 and 2 [9]. In response to UV (280 nm) illumination phosphorylation of AKT and ERK1/2 is not detectable upon activation with EGF. Given the observed upregulation of p21WAF1 in our previous work [9], which is a cyclin dependent kinase inhibitor, we must assume that UV illumination causes the cells to arrest the cell cycle in G1 phase, which would be beneficial in inhibiting the proliferative potential of EGFR overexpressing cells. Adachi S. et al have also recently shown that UV irradiation can induce evasion of colon cancer cells from stimulation of epidermal growth factor [58]. They report that UV (254 nm) caused inhibition of cell survival and proliferation, concurrently inducing the decrease in cell surface EGFR and subsequently its degradation. Furthermore, the same team has reported that UV (254 nm) significantly inhibited platelet-derived growth factor PDGF-BB-induced phosphorylation of Akt on pancreatic cancer cells [59]. Several reports exist, describing how UV light can activate the EGF receptor hence activating the AKT and MAPK pathway (43–50 from ref. 9). These observations are in contrast to our results. Wan et al. have reported that UVB irradiation stimulated PI 3-kinase activity in human skin *in vivo* [60]. UV also stimulated phosphorylation of the downstream AKT effectors, S6 kinase and BAD. Inhibitors of EGFR and PI 3-kinase blocked UV-induced phosphorylation of BAD, suggesting that EGFR mediates UV-activated cell survival pathway. They concluded that both positive and negative roles for UV activation of the PI 3-K/AKT pathway in human skin can be envisioned. The PI 3-K/AKT pathway likely plays a critical role in balancing UV-induced apoptotic signals, thereby preventing widespread skin cell death. Conversely UV activation of the PI 3-K/AKT pathway may enhance survival of mutated cells, thereby promoting skin cancer, as has been found in several other types of cancer. Cao et al. [61] have concluded that UV (>290 nm, UVB/UVA2 source peaking we believe at 445 nm) induces multiple signalling pathways mediated by EGFR trans-activation leading to possible maturation, apoptosis and survival, and EGFR activation protects against UV-induced apoptosis in cultured mouse dendritic cells. Authors also report that most of the UV responses are mediated by the production of reactive oxygen species (ROS) and can be blocked by antioxidants. UVC is reported to induce rapid and transient expression of early growth response-1 gene (Egr-1) encoding a transcription factor that plays a role in cell survival. UVC irradiation causes tyrosine phosphorylation of EGFR in mouse NIH 3T3 fibroblasts and HC11 mouse mammary cells [50]. Possible reasons for the reported discrepancies could be found in the wavelength used in the experiments (from 254 nm to 445 nm), in the illumination power per unit of illuminated area and in the type of cells used (mutated vs non-mutated cells). Unfortunately these values are not accurately reported in all papers in order to make comparisons possible.

The present paper has provided unequivocal evidence for a UVB induced structural modification in EFGR, resulting in structural changes in the EGF binding site and loss of function. Interestingly, these changes were observed at a photonic power level approximately in the same order of magnitude as the integrated power in the solar UVB range. Therefore, it is reasonable to believe that UVB possesses therapeutic properties, especially towards skin and other superficial types of cancer. Clearly there must be a threshold irradiance level and illumination time, below which UVB light can be used for cancer treatment. Our most recent studies on the on the UV (280 nm) effects on EGF-EGFR activation of A549 lung carcinoma cells overexpressing EGFR (paper in preparation) confirm the hypothesis raised in this study that 280 nm light will most likely also change the structure/function of the extracellular domain of EGFR when present in the cell surface of cancer cells overexpressing EGFR, preventing this way EGF-EGFR binding and activation.

## Materials and Methods

### Structure Analysis

The crystallography data used for the display of the 3D protein structure (Figure 2) was extracted from 1ivo.pdb (extracellular domain of human epidermal growth factor receptor complexed with EGF, 3.3 Å resolution [1]). Accelrys Discovery Studio Visualizer 3.5 was used for displaying the 3D structure. Distances between protein residues were obtained by using the monitor tool in the program to determine the distance between atoms in the 3D structure. The fraction of disulphide bridges was calculated as the number of disulphide bridges found in a protein divided by the protein sequence length (number of amino acids) x 100 (Figure 3). The pdb dataset used in order to display the dependence of the average fraction of disulphide bridges on the protein chain length has been published previously by our group [28].

### Fluorescence Studies

Fluorescence studies were carried out in order to analyze the effects of 280 nm excitation on the structure of the extracellular domain of human EGFR (sEGFR). sEGFR was purchased from Speed Biosystems (YCP1031). This protein was expressed in human embryonic kidney (HEK) 293 cells. The amino acid sequence corresponds to sEGFR (Leu25-Ser645) with a C-terminal polyhistidine (His) tag. The protein was dissolved directly in 10 mM Sodium Phosphate Buffer (NaPB) at pH 7.5 (stock solutions). Milli-Q water with conductivity below 0.2 µS.cm^−1^ was used. sEGFR concentration was determined by Abs^280nm^ using a molar extinction coefficient of 60,000 M^−1^cm^−1^ estimated using the bioinformatic tool ProtParam (Expasy, [62], entry: sequence of extracellular domain of EGFR (Leu25-Ser645) with a C-terminal polyhistidine (His) tag). Stock solutions were used within 2-3 days after dissolution in buffer and was kept desiccated and protected from light at 4°C. Before each experiment the stock solution was diluted to 3.9 µM. Unless stated otherwise, this was the concentration used in all the fluorescence studies.

### Steady state studies

UVB illumination of sEGFR was carried out in a ChronosBH spectrometer (ISS) with a T-configuration, using a 300-W Xenon arc lamp coupled to a monochromator. Excitation and emission slits were set to 4 nm and 8 nm, respectively. Lamp power at the sample location was 89 µW (at 280 nm). The illumination spot was 0.35 cm^2^. Irradiance was 2.5 W.m^−2^. The temperature of the solution was kept at 20°C using a Peltier element at the cuvette holder location. One hundred L of sEGFR stock solution was placed in a quartz cuvette (2 mm×10 mm×6 mm excitation volume) and excited at 280 nm during a maximum time of 75 min. Samples were illuminated through the 2 mm×6 mm window and fluorescence collected from the 10 mm×6 mm window. A fresh sample was used for each illumination session. Emission spectra were acquired with 280 nm, 295 nm, 320 nm and 360 nm excitation. Excitation spectra were recorded with the emission fixed at 320 nm, 334 nm, 350 nm, and 400 nm. Each of the previous spectra was recorded before illumination (0 min), and after 15 min, 30 min, 45 min, 60 min and 75 min of illumination. The same emission and excitation spectra were acquired for the buffer. The emission and excitation intensity values obtained were corrected in real-time for oscillations in the intensity of the excitation lamp. Buffer Raman signal was subtracted from each emission spectrum. The 350 nm fluorescence emission intensity upon continuous 280 nm excitation was recorded during 75 min of illumination.

#### Fluorescence based protein thermal unfolding studies

Trp emission is sensitive to the extent of solvent accessibility. The more solvent accessible Trp is the more red shifted its fluorescence emission will be. Therefore, Trp emission is usually used as a probe for protein conformational changes and can be used to determine the melting temperature of the protein.

The 330 nm fluorescence emission intensity (exc. 295 nm) of a fresh sEGFR sample (3.49 µM) and of a 280 nm illuminated for 75 min sEGFR sample (3.07 µM) was monitored from 4 °C to 90 °C. The sEGFR sample was continuously illuminated at 280 nm and as it was previously described. The heating rate was 1°C/min. A point was acquired every minute. After reaching 90 °C the protein samples were cooled from 90 °C to 4 °C at a cooling rate of 1 °C/min and the fluorescence intensity at 330 nm was monitored (exc. 295 nm).

#### Time resolved studies

A sEGFR sample was continuously illuminated at 280 nm during 75 min. The experimental set-up and parameters used for UVB illumination were the same as previously described. As negative control, a freshly prepared sEGFR sample was kept in the dark for 75 min. After UV illumination or dark period (negative control), the sample was kept in the cuvette and lifetime measurements were carried out at magic angle conditions using TCSPC method. A 283 nm light emitting diode from ISS (FWHM  = 9 nm) was used to excite the samples. The fluorescence emission at magic angle (54.7°) was counted by a GaAs detector (Hamamatsu H7422P-40), and a 300 nm long-pass filter (Semrock) was employed in order to stray light. A solution of Ludox in Millipore water was used as a reference sample. The instrument response function for this system is 180 ps.

### Detection of thiol groups' concentration formed upon UV illumination of sEGFR

Preparation of sEGFR solutions and buffers was carried as described in the previous section. One hundred L of sEGFR work solution (3.9 µM) was placed in a quartz macro cuvette (1 cm path length) and illuminated at 280 nm during 75 min. 280 nm illumination was carried out as described in the previous section. In parallel, 100 µL of the same sEGFR work solution was kept in the dark for 75 min (negative control sample, dark NC). Ellman's assay was used in order to detect free thiol groups formed upon UVB induced disruption of disulphide bridges in sEGFR [19], [21], [22], [63]. Ellman's reagent, 5,5′-dithiobis-2-nitrobenzoic acid (DTNB) was purchased from Molecular Probes (product D8451, Life Technologies, Naerum, Denmark). One hundred mM stock solution was prepared in DMSO and stored at 4°C. After illumination, 150 µL of 2 times diluted illuminated or control sEGFR solution was mixed with an excess of DTNB (1.5 µL of 100 mM stock solution). The molar ratio DTNB/sEGFR was 5.13. Four minutes after mixing the two components (sample kept in the dark), the absorbance intensity at 412 nm was measured in a UV/Visible spectrophotometer (Shimadzu Corporation, 3. Kanda-Nishikicho 1-chome, Chiyoda-ku, Tokyo 101–8448, Japan), using a 1 cm path length quartz cuvette. The product of the reaction is the 2-nitro-5-thiobenzoate ion (TNB^2−^), which absorbs at 412 nm. Abs_412nm_ is proportional to the amount of thiol groups present in solution. The concentration of thiol groups was determined using an extinction molar coefficient for TNB^2−^ of 14150 M^−1^.cm^−1^ at 412 nm [63].

### Circular dichroism studies

The circular dichroism spectrum of a fresh sEGFR sample and of a 280 nm illuminated for 75 min sEGFR sample was acquired in order to monitor the UVB loss of secondary structural elements. Preparation of sEGFR solutions and buffers was carried as previously described. One hundred µL of sEGFR work solution (3.9 µM) was placed in a quartz macro cuvette (1 cm path length) and illuminated at 280 nm during 75 min. The experimental set-up, conditions and parameters used during the illumination procedure were the same as previously described. Immediately after the illumination, 200 µL of 3 times diluted illuminated sEGFR solution was placed in a quartz microcuvette with a path length of 0.1 cm and cooled to 4°C using a Peltier element at the cuvette holder's location. Afterwards, a far-UV CD spectrum was recorded. Temperature was kept constant at 4°C during the measurement. The same was done for a fresh sEGFR sample. Far-UV CD spectra (190–240 nm) were acquired using the following parameters: 1.0 nm band width, resolution 0.5 nm, 4 accumulations, scan speed 20 nm/min, sensitivity high, 16 s response time. Far-UV CD spectrum was also acquired for the buffer. The buffer signal was subtracted from all spectra. The measurements were carried out on a JASCO J-815 CD spectrometer (JASCO Corporation, Ishikawa-cho Hachioji-shi, Tokyo, Japan).

#### Circular dichroism based protein thermal unfolding studies

The ellipticity intensity at 220 nm of a fresh sEGFR sample and of a 280 nm illuminated for 75 min sEGFR sample (both solutions 3.9 µM) was continuously monitored from 4°C to 90°C. The experimental set-up, conditions and parameters used during the illumination procedure were the same as described in the previous sections. The heating rate was 1 °C/min. A point was acquired every minute. Far-UV CD spectra (190–240 nm) were acquired at 4°C (before heating) and at 90°C. Afterwards the protein samples were cooled from 90°C to 4°C at a cooling rate of 1 °C/min and the ellipticity intensity at 220 nm was monitored every minute. At the end, far-UV CD spectra (190–240 nm) were acquired at 4°C. Far-UV CD spectra (190–240 nm) were recorded using the parameters described in the previous section.

### Immunoassay

A binding immunoassay was carried out in order to analyze the effects of 75 min of UV illumination at 280 nm on the structure of the EGF-sEGFR binding site. The primary mouse monoclonal antibody anti-EGFR neutralizer antibody LA1 from Milipore (05–101), which competes with EGF and TGF-α for binding EGFR [54] was used.

For each experiment, 1.4 µg of fresh non-illuminated protein sample, 1.4 µg of a 280 nm illuminated sample for 75 min and 1.4 µg of a protein sample that has been in the dark for 75 min (negative control, NC) were analyzed by Western blot. The experimental set-up, conditions and parameters used during UV illumination procedure were the same as described in the previous sections. Samples were resolved by reduced sodium dodecyl sulfate polyacrylamide gel electrophoresis (SDS-PAGE), 7.5% polyacrylamide from Bio-Rad (4561023) and transferred to a 0.2 µm nitrocellulose membrane from Whatman (10402495). Then, the membrane was probed with 11000 dilution of LA1 primary antibody followed by incubation with 15000 dilution of goat anti-mouse horseradish peroxidase (HRP)-conjugated secondary antibody from Santa Cruz Biotech (SC-2005). Immune complexes were visualized on nitrocellulose by enzyme-linked enhanced chemiluminescence (ECL) from GE Healthcare (RPN2232) and detected using the CCD camera of G:Box chemi XT4 controlled by Genesys software from Syngene. Band intensities were quantified using the gel analysis software GeneTools from Syngene. The immunoassay was carried out twice.

### Data Analysis

All data analysis, plotting and fitting procedures were done using Origin 8.1.

#### Emission Spectra and Excitation Spectra

Emission Spectra (280 nm, 295 nm, 320 nm, and 360 nm excitation) were first smoothed using a 12 points adjacent averaging. Excitation Spectra (emission fixed at 334 nm and 400 nm excitation) were smoothed using a 5 points adjacent averaging. All fluorescence spectra obtained were first Raman corrected by subtracting the spectra recorded for the buffer in solution. Normalized emission and excitation spectra were obtained by dividing each data point by the maximum intensity value in each spectrum.

#### Fitting Procedures

*Fluorescence emission kinetic trace at 350 nm upon 280 nm exc*: The fluorescence emission kinetic trace (em. 350 nm, Fig. 4A) was fitted using a bi-exponential function *F(t) =  y_0_ + C*1.*e^-k^* ^1.*t*^ *+ C*2.*e^-k^* ^2.*t*^. *F(t)* is the fluorescence emission intensity at 350 nm (a.u.) at 280 nm excitation time *t* (min), *y_0_*, *C*1 and *C*2 are constants and *k*1 *and k*2 is the rate constant of fluorescence emission intensity decrease (min^-1^). *y_0_* value was fixed to 0.

*Fluorescence based protein thermal unfolding studies*: The fluorescence emission intensity thermal curves (Fig. 5) were first smoothed using a 5 points adjacent averaging. The fluorescence emission intensity values were afterwards normalized by dividing each value by the initial 330 nm emission intensity value. The curve corresponding to the non-illuminated sEGFR sample (non illum.) was then fitted using a modified Boltzmann function:
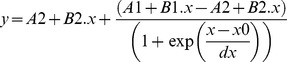
where *y* (a.u.) is the 330 nm fluorescence emission intensity at the temperature *x* (°C), *A*1, *B*1, *A*2, *B*2 and *dx* are constants. The fitting parameter *x*0 (°C) corresponds to the inflection point of the Boltzmann curve and the corresponding temperature is the temperature of mid-transition determined upon probing Trp fluorescence emission.

*Fluorescence lifetimes*: The fluorescence decay was analyzed by an ISS routine based on the Marquardt least-squares minimization. The governing equations for the time-resolved intensity decay data were assumed to be a sum of discrete exponentials as in: 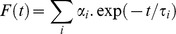
where *F*(*t*) is the intensity decay, α*_i_* is the amplitude (pre-exponential factor), *τ_i_* the fluorescence lifetime of the *i*-th discrete component, and ∑ α*_i_*  = 1.0.

The fractional intensity *fi* of each decay time is given by:
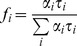
and the mean lifetime is:
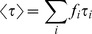



#### Circular dichroism based protein thermal unfolding studies

Only the data values obtained above 10°C were then considered for analysis. The ellipticity intensity values were first smoothed using a 12 points adjacent averaging. The data values were then normalized by dividing each ellipticity intensity value by the initial ellipticity intensity value (i.e. first data point above 10°C). The curve corresponding to the non-illuminated sEGFR sample (non illum.) was then fitted using a Boltzmann function:
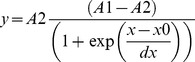
where *y* (a.u.) is the 220 nm ellipticity intensity values at the temperature *x* (°C), *A*1, *A*2, and *dx* are constants. The fitting parameter *x*0 (°C) is the inflection point of the Boltzmann curve and its value corresponds to the temperature of mid-transition of the curve. The fitting was done for the interval 57.12–69.48°C, which comprises the thermal transition part of the curve.
