# Correction: Modulating the Structure of EGFR with UV Light: New Possibilities in Cancer Therapy

**DOI:** 10.1371/journal.pone.0144790

**Published:** 2015-12-07

**Authors:** Manuel Correia, Viruthachalam Thiagarajan, Isabel Coutinho, Gnana Prakash Gajula, Steffen B. Petersen, Maria Teresa Neves-Petersen

An affiliation for the 6^th^ author is not included. Maria Teresa Neves-Petersen is affiliated with: **2** BioPhotonics Group, Department of Nanomedicine, International Iberian Nanotechnology Laboratory (INL), Braga, Portugal and **6** Department of Clinical Medicine, Aalborg University, Søndre Skovvej 15, 9000 Aalborg, Denmark.
